# Effects of 25-Min Nap Opportunity during Ramadan Observance on the 5-m Shuttle Run Performance and the Perception of Fatigue in Physically Active Men

**DOI:** 10.3390/ijerph17093135

**Published:** 2020-04-30

**Authors:** Hsen Hsouna, Omar Boukhris, Khaled Trabelsi, Raouf Abdessalem, Achraf Ammar, Khadijah Irandoust, Morteza Taheri, Nizar Souissi, Roy Jesse Shephard, Sergio Garbarino, Nicola Luigi Bragazzi, Hamdi Chtourou

**Affiliations:** 1 Activité Physique, Sport et Santé, UR18JS01, Observatoire National du Sport, Tunis 1003, Tunisie; hsen.hsouna92@gmail.com (H.H.); omarboukhris24@yahoo.com (O.B.); raoufabdesalem18@gmail.com (R.A.); n_souissi@yahoo.fr (N.S.); h_chtourou@yahoo.fr (H.C.); 2 Institut Supérieur du Sport et de L’éducation Physique de Sfax, Université de Sfax, Sfax 3000, Tunisie; trabelsikhaled@gmail.com; 3 Research Laboratory: Education, Motricité, Sport et Santé, EM2S, LR19JS01, High Institute of Sport and Physical Education of Sfax, University of Sfax, Sfax 3000, Tunisia; 4 Institute of Sport Science, Otto-von-Guericke University Magdeburg, 39104 Magdeburg, Germany; Ammar.achraf@ymail.com; 5 Department of Sport Sciences, Imam Khomeini International University, Qazvin 34148-96818, Iran; irandoust@soc.ikiu.ac.ir (K.I.); taheri_morteza@yahoo.com (M.T.); 6 Faculty of Kinesiology and Physical Education, University of Toronto, Toronto, ON M5S 1A1, Canada; royjshep@shaw.ca; 7 Department of Neuroscience, Rehabilitation, Ophthalmology, Genetics, Maternal and Child Health (DINOGMI), University of Genoa, 16132 Genoa, Italy; sgarbarino.neuro@gmail.com; 8 Department of Health Sciences (DISSAL), Postgraduate School of Public Health, University of Genoa, 16132 Genoa, Italy; 9 Department of Mathematics and Statistics, Laboratory for Industrial and Applied Mathematics (LIAM), York University, Toronto, ON M3J 1P3, Canada

**Keywords:** fasting, sleep, exercise, fatigue, nap

## Abstract

We aimed to investigate the effects of a 25-min nap opportunity on physical performance during the 5-m shuttle run test (5mSRT), feelings (i.e., evaluated by the feeling scale), attention (i.e., evaluated by the digit cancellation test) and the perception of fatigue (i.e., recorded by the rating of perceived exertion (RPE)) during Ramadan observance. Twelve physically active men (age: 21.1 ± 3.2 yrs, height: 1.76 ± 0.05 m, body-mass: 71.2 ± 9.3 kg) voluntarily participated in five test sessions: 15 days before Ramadan (BR), the first 10 days of Ramadan (FR), the last 10 days of Ramadan (ER), 10 days after Ramadan (10AR) and 20 days after Ramadan (20AR). During each test session, participants performed the digit cancellation test, a 5-min standard warm-up, the 5mSRT (6 × 30-s with 35-s intervals-between) and the rating of perceived exertion (RPE) after no-nap (N0) and 25-min nap opportunity (N25) conditions. Participants also completed the Pittsburgh Sleep Quality Index (PSQI) during each period. The total distance covered during the 5mSRT did not differ significantly before, during or after Ramadan, but was significantly greater after N25 compared to N0 at 10AR (687.5 ± 23.0 m vs. 725.6 ± 41.1 m; *p* = 0.018) and 20AR (698.3 ± 19.8 m vs. 742.6 ± 58.3 m; *p* = 0.003). The attention scores were higher after N25 in comparison with N0 at 10AR (*p* = 0.04) and 20AR (*p* = 0.02). RPE scores were not significantly different between N25 and N0 conditions. Feelings scores were higher after N25 compared to N0 during both FR (*p* = 0.007) and 20AR (*p* = 0.04). A significant deterioration of sleep quality was recorded during Ramadan (i.e., PSQI scores were significantly higher during and after compared to BR (*p* < 0.0005)). A 25-min nap opportunity was beneficial for physical and cognitive performance after Ramadan observance; however, any effect is insufficient to show significant beneficial impacts during Ramadan.

## 1. Introduction

The optimization of the recovery process to reach the peak performance is a primary goal for athletes, coaches and scientists [1]. It has been suggested that good-quality sleep assists the recovery process during both training and competition [2]. However, athletes are frequently exposed to various situations that may impair sleep–wakefulness patterns during the observance of Ramadan [3,4,5].

During the 29 or 30 days of Ramadan observance, healthy adult pubescent Muslims must abstain from eating, drinking and sexual relations from sunrise to sunset [6]. This month is characterized by perturbations on sleep patterns [3,4,5] in addition to disturbances of food and fluid intake [5,7,8]. Although some studies have not reported any significant changes in physical fitness level [9,10,11] or cognitive function [12] during Ramadan, a decrement in physical performance has been reported in both athletes [13,14,15,16] and physically active men [17,18]. Additionally, decreases in cognitive performance have been reported in some groups of physically active young men [19] and athletes [20].

Previous studies have suggested that physical performance was impaired during Ramadan because of negative effects on both physiological and cognitive functions [14,15,17,21,22]. Changes in patterns of food and fluid intake [7] and sleep–wake behaviors [3,23] may be the principal factor underlying the decrements of physical and cognitive performance. In one study, Ramadan observance also negatively affected fatigue and sleep as estimated by the Hooper questionnaire in soccer players, without any impact on perceptions of stress or muscle soreness [24]. In contrast, Boukhris et al. [19] reported that Ramadan observance did not affect the Hooper estimation of stress, sleep, fatigue and muscle soreness.

Clearly, the implementation of legitimate tactics that may help athletes cope with any drop in performance and behaviors is warranted. Rebai et al. [25] suggested that two weeks of tapering (i.e., reducing the resistance training volume) during Ramadan limited fatigue and helped maintain the same physical performance as before Ramadan. Several previous studies have also reported changes in sleep–wakefulness patterns during Ramadan (e.g., a reduction in total sleep time, sleep fragmentation, and a shift in the sleep–wake cycle) [3,26,27,28], so that it seems important to include a daytime nap to counteract sleep perturbations. In support of this suggestion, Waterhouse et al. [29] found that short-duration naps helped mitigate negative effects on cognitive and physical performance. A 30 min of nap had a beneficial effect on 20-m sprint time after one night of sleep deprivation. Boukhris et al. [30] and Abdessalem et al. [31] also reported that even after a normal sleep, a 25-min nap opportunity improved performance and reduced fatigue during the 5-m shuttle run test (5mSRT). Additionally, Hsouna et al. [32] reported that after a night of normal sleep, a 45-min nap opportunity improved performance during the 5-jump test and increased attention. Likewise, Daaloul et al. [33] reported that a 30-min nap enhanced cognitive outcomes, and argued that a nap opportunity was an effective tactic to overcome the cognitive and physical deteriorations in performance caused by either sleep loss or the fatigue induced by exhaustive training.

Surprisingly, no previous study has investigated the effect of a short-duration nap opportunity on physical and cognitive performance during Ramadan. Therefore, the aim of the present study was to investigate the effect of a 25-min nap opportunity on physical performance during the 5mSRT, attention, feelings and subjective perceptions of fatigue in physically active men during Ramadan observance. We hypothesized that a 25-min nap opportunity would improve physical and cognitive performance and reduce perceptions of fatigue during Ramadan observance.

## 2. Materials and Methods

### 2.1. Participants

The necessary minimum sample size was calculated a priori using the software G*power [34], using procedures suggested by Beck [35]. Values for α were set at 0.05 and for power at 0.95. Based on an earlier study of Herrera et al. [36] and discussions between the authors, effect sizes were estimated at 0.46. To reach the desired power, data from at least eleven participants were needed to minimize the risk of incurring a type 2 statistical error.

Fifteen participants were recruited. Three participants were excluded as they did not initiate the sleep. Twelve physically active men (age: 21.1 ± 3.2 yrs, height: 1.76 ± 0.05 m, body-mass: 71.2 ± 9.3 kg) voluntarily participated in the present study. After receiving a full description of the protocol, potential risks, and benefits, they each gave their written consent to participate in the present study. All were non-smokers, without pathological sleep disorders, they did not consume alcohol and did not work/study during the entire period of the investigation. They were not members of formal sports teams, but they practiced a moderate amount of physical activity (i.e., typically fast walking and jogging) for ≈1 h/day, 3 days/week. The usual time of exercising was 17h00, and this was not modified in volume (i.e., ~8 km of jogging in 1 h) or timing during Ramadan. They were living at home and received no-specific advice on methods of minimizing the effects of Ramadan observance.

The present study was conducted according to the Code of Ethics for human experimentation, the Declaration of Helsinki [37] and the protocol was fully approved by the Research Ethics Committee before the commencement of the assessments.

### 2.2. Experimental Design

The study was carried out in Tunisia when the fasting duration was ~16 h. Averages for temperature and relative humidity were: 28 °C and 50% before Ramadan, 32 °C and 49% during the last ten days of Ramadan, and 31 °C and 47% during the 20AR.

After a familiarization session, participants visited the laboratory on five separate occasions: 15 days before Ramadan (BR), the first 10 days of Ramadan (FR), the last 10 days of Ramadan (ER), 10 days after Ramadan (10AR) and 20 days after Ramadan (20AR). During each period, participants undertook two test sessions in a random order, i.e., a no-nap opportunity (N0) and a 25 min of nap opportunity (N25), with at least 72 h in-between. After the arrival of the participants to the laboratory, 15 min was allowed for them to become accustomed to their new place of sleep. At 13:45h, participants were asked to be prepared for the new place and were given a 25-min of nap opportunity in dark and quiet sleeping rooms until 14h00. From then until 17h00, participants in both during N0 and N25 conditions spent the time in habitual activities (e.g., watching TV, playing video games), but they undertook no physical activity. At 17h00, they completed the digit cancellation test of attention. Then, after 5 min of a standardized warm-up consisting of two minutes of easy running followed by three minutes of dynamic exercises performed over a 10-m distance (i.e., lateral shuffles, internal-external hip rotations, high knees, heel kicks, straight leg marches and lunges), they responded to the feelings scale (FS) and performed the 5mSRT (6 × 30-s with 35-s in-between) with the estimation of the rating perceived exertion (RPE) after each repetition of the test. During each visit, they also completed the Pittsburgh Sleep Quality Index (PSQI) and recorded the amount and type of food and fluid consumed.

### 2.3. The 5-m Shuttle Run Test

The test consisted of six repetitions of 30 s maximal shuttle sprints over distances of 5 m, 10 m, 15 m, and 20 m, alternatively, with intervening recovery period of 35 s. Due to these characteristics, this exercise was considered as a test of anaerobic endurance. This exercise is widely used for physical quality assessment and for the training program [30]. For the full description of the 5mSRT see Boukhris et al. [30].

The following parameters were calculated:

- Total distance (TD) (m) = Sum of distances covered during the 6 × 30-s shuttles
- Best distance (BD) (m) = The greatest distance covered during the 6 × 30-s shuttles
- Fatigue index (FI) (%) = {([(shuttle 1 + shuttle 2)/2] – [(shuttle 5 + shuttle 6)/2])/[(shuttle 1 + shuttle 2)/2]} × 100

### 2.4. Rating of Perceived Exertion Scale (RPE)

The RPE scale that was used ranged from “0” (very very light) to “10” (very very hard) [38]. Immediately after the completion of each 30-s repetition, participants were shown the RPE scale and asked to assess their feeling of exertion on completion of the task. The RPE value used in the statistical analyses was the mean score during the 5mSRT calculated as:

RPE (AU)=Σ RPE during the 5m shuttle run test/ Number of repetitions

### 2.5. The Digit-Cancellation Test

As previously used by Hsouna et al. [32], participants performed the digit-cancellation test for one minute, deleting target numbers (i.e., numbers composed by three grouped digits) on a sheet of randomly arranged possibilities. As indicated in the study of Hsouna et al. [32], this test was used for the assessment of various aspects, e.g., processing speed of information, attention focus and executive function. The sum of the correctly deleted numbers was registered for analysis.

### 2.6. Feelings Scale

It is common to experience changes in mood while exercising. Some individuals find exercise pleasurable, whereas others find it to be unpleasant [39]. The FS is an 11-point, single-item, bipolar scale (scores of “−5” as “very strong feeling of displeasure” to “+5” as “very strong feeling of pleasure”).

### 2.7. Dietary Intake Analysis

Participants recorded all meals consumed throughout the experimental period, noting both the amounts and types of food and fluid consumed. They were also interviewed by an experienced nutritionist. Findings were analyzed using the software program Bilnut (NutrisoftBilnut: Food Survey Program version 2.01) and the food-composition tables of the Tunisian National Institute of Statistics (1978).

### 2.8. The Pittsburgh Sleep Quality Index

The subjective sleep quality was assessed using the PSQI questionnaire [40]. This comprises 19 questions, grouped into seven components: sleep duration, sleep quality, sleep latency, sleep efficiency, sleep disturbances, the use of sleeping medications, and daytime dysfunction. The total score of the PSQI ranges from “0” to “21”, where “0” indicates “no difficulty” and “21” indicates “severe difficulties”.

### 2.9. Statistical Analyses

All statistical tests were processed using STATISTICA 10.0 Software (Stat-Soft, Maisons-Alfort, Paris, France). Means and SE (standard errors) were calculated for each variable.

The normality of the data was assessed using the Shapiro Wilk’s test. When the distribution of the data was normal, a two-factor ANOVA (Period × Nap) was performed for TD during the 5mSRT and the number of correct answers estimated during the digit cancellation test (attention). A one-factor ANOVA (Period) was performed for PSQI, total energy intake and carbohydrate data. When appropriate, significant differences between means were tested using the Bonferroni post hoc test.

When the Shapiro-Wilk test was significant (*p* < 0.05), the Friedman test was performed for BD and FI recorded during the 5mSRT, the feelings scale scores, the RPE scores, and the protein and the lipid consumption. Effect sizes were calculated as partial eta-squared (*η_p_^2^*) for the ANOVA analysis to determine the magnitude of the change and were assessed as: <0.2 = trivial, 0.2–0.6 = small, 0.6–1.2 = moderate, 1.2–2.0 = large, and >2.0 = very large [41]. When the data was skewed, the effect size was estimated by Kendall’s coefficient of concordance.

Significance was accepted for all analyses at the level of *p* < 0.05.

## 3. Results

### 3.1. The 5-m Shuttle Run Test

#### Total Distance

**Figure 1:**
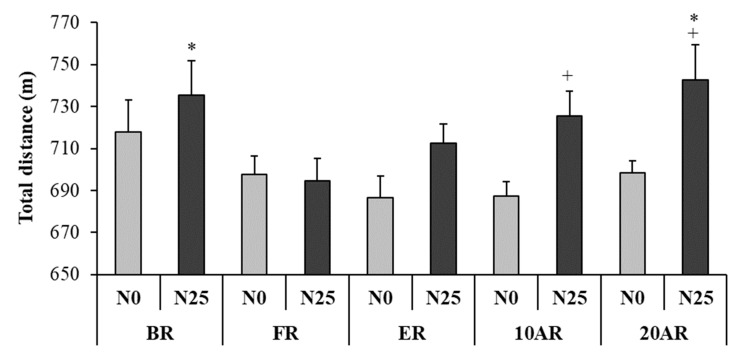
Total distance (mean ± SE) during the 5-m shuttle run test recorded 15 days before Ramadan (BR), during the first 10 days of Ramadan (FR), the last 10 days of Ramadan (ER), 10 days after Ramadan (10AR) and 20 days after Ramadan (20AR) after a no-nap (N0) and a 25-min of nap opportunity (N25) conditions. +: significant difference in comparison with N0; *: significant difference in comparison with FR for N25.

The repeated measures ANOVA showed significant main effects of Period (F = 4.23; *p* = 0.005; *η_p_^2^* = 0.28) and Nap (F = 10.22; *p* = 0.008; *η_p_^2^* = 0.48) and a significant interaction Period × Nap (F = 3.48; *p* = 0.01; *η_p_^2^* = 0.24) (Figure 1). During N25, Bonferroni post-hoc testing revealed a significant decrease of TD during the 5mSRT at FR compared to BR (717.9 ± 52.5 m vs. 694.8 ± 10.4 m; *p* = 0.008) and 20AR (735. 3 ± 15.2 vs. 694.8 ± 10.4 m; *p* = 0.008). There was also a significant increase of TD during 5mSRT after N25 compared to N0 at 10AR (687.5 ± 23.0 m vs. 725.6 ± 41.1 m; *p* = 0.018) and 20AR (698.3 ± 19.8 m vs. 742.6 ± 58.3 m; *p* = 0.002).

### 3.2. Best Distance

**Figure 2:**
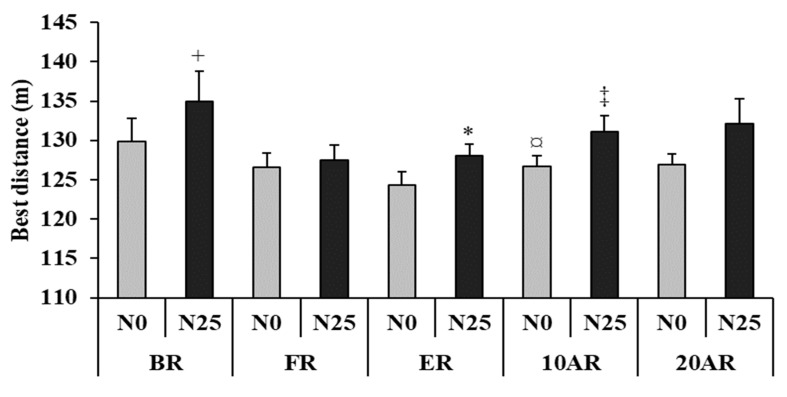
Best distance (mean ± SE) during the 5-m shuttle run test recorded 15 days before Ramadan (BR), during the first 10 days of Ramadan (FR), the last 10 days of Ramadan (ER), 10 days after Ramadan (10AR) and 20 days after Ramadan (20AR) after a no-nap (N0) and a 25-min of nap opportunity (N25) conditions. +: significant difference in comparison with N0; *: significant difference in comparison with FR for N25; ‡: Significant difference in comparison with FR for N25; ¤: Significant difference in comparison with ER for N0.

The Friedman test revealed a significant main effect (test = 19.35, *p* = 0.02, Kendall’s W = 0.18) (Figure 2). During N0, pairwise comparisons showed that BD during the 5mSRT was significantly lower at ER compared to 10AR (124.3 ± 5.9 m vs. 126.7 ± 5.0 m; *p* = 0.025) and 20AR (124.3 ± 5.9 m vs. 126.9 ± 4.8 m; *p* = 0.028). During N25, BD during the 5mSRT was significantly lower (i) at ER compared to BR (128.0 ± 5.3 m vs. 135.0 ± 13.1 m; *p* = 0.042) and 10AR (128.0 ± 5.3 m vs. 131.1 ± 7.0 m; *p* = 0.028) and at FR compared to 10AR (127.5 ± 6.7 m vs. 131.1 ± 7.0 m; *p* = 0.036). Additionally, there was a significant increase of BD after N25 compared to N0 at BR (129.8 ± 10.3 m vs. 135.0 ± 12.1 m; *p* = 0.043) and 10AR (126.7 ± 5.0 m vs. 131.1 ± 7.0 m; *p* = 0.037).

### 3.3. Fatigue Index

**Figure 3:**
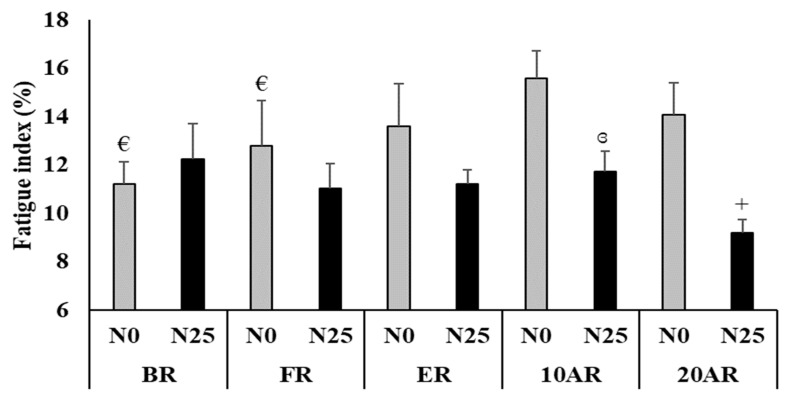
Fatigue (means ± SE) during the 5-m shuttle run test recorded 15 days before Ramadan (BR), during the first 10 days of Ramadan (FR), the last 10 days of Ramadan (ER), 10 days after Ramadan (10AR) and 20 days after Ramadan (20AR) after a no-nap (N0) and a 25-min of nap opportunity (N25) conditions. +: significant difference in comparison with N0; €: Significant difference in comparison with 10AR for N0; ɞ: Significant difference in comparison with 20AR for N25.

The Friedman test showed a significant effect (test = 22.59, *p* = 0.007, Kendall’s W = 0.21) (Figure 3). During N0, the Wilcoxon test showed a significant increase of FI during the 5mSRT at 10AR in comparison with BR (*p* = 0.01) and FR (*p* = 0.04). The FI was significantly lower after N25 compared to N0 at 10AR (*p* = 0.009) and 20AR (*p* = 0.04).

### 3.4. Rating of Perceived Exertion

The repeated measures analysis of variance showed a significant effect of Nap (F = 8.64; *p* = 0.01; *η_p_^2^* = 0.44) (Table 1). The post-hoc test revealed a significant increase of attention after N25 in comparison with N0 at 10AR (*p* = 0.04) and 20AR (*p* = 0.02). Additionally, after N25, the attention was greater at 20AR compared to ER (*p* = 0.02).

### 3.5. Rating of Perceived Exertion

The Friedman test showed a significant effect on RPE scores (test = 34.46, *p* < 0.0005, Kendall’s W = 0.32) (Table 1). During N0, post-hoc comparisons revealed a significant decrease of RPE scores during BR in comparison with ER (*p* = 0.03), 10AR (*p* = 0.01) and 20AR (*p* = 0.002). Additionally, after N25, there was a significant decrease of RPE scores during BR in comparison with FR (*p* = 0.003), ER (*p* = 0.008), 10AR (*p* = 0.005) and 20AR (*p* = 0.003).

### 3.6. Feelings Scale

There were no significant effect on feelings scale scores after the warm-up (test = 9.95, *p* = 0.35, Kendall’s W = 0.09) (Table 1). However, there was a significant effect on feelings scale scores after the 5mSRT (test = 23.46, *p* = 0.005, Kendall’s W = 0.22). The feelings scale scores were significantly higher after N25 compared to N0 during FR (*p* = 0.007) and 20AR (*p* = 0.04).

### 3.7. Dietary Intake

No significant changes in caloric intake (F = 1.84; *p* = 0.13; *η_p_^2^* = 0.14) or protein ingestion (test = 1.77, *p* = 0.77, Kendall’s W = 0.03) were reported (Table 2), but there were significant changes for carbohydrates (F = 6.40; *p* = 0.0003; *η_p_^2^* = 0.37) and lipids (test = 14.15, *p* = 0.006, Kendall’s W = 0.29) (Table 2). Carbohydrate intake was significantly higher during 10AR and 20AR in comparison with FR (*p* = 0.006 and *p* = 0.007, respectively) and ER (*p* = 0.01 and *p* = 0.01, respectively). Lipid intake was significantly higher during ER in comparison with 10AR (*p* = 0.009) and 20AR (*p* = 0.009).

### 3.8. The PSQI Questionnaire

Statistical analysis revealed a significant effect of periods (F = 24.61; *p* < 0.0005; *η_p_^2^* = 0.69) (Table 3). The post-hoc test showed a significant deterioration of the sleep quality during Ramadan. Total PSQI scores were significantly higher during (*p* < 0.0005) and after (*p* < 0.0005) Ramadan in comparison with BR. The PSQI questionnaire also indicated a significant perturbation of the subjective sleep quality (test = 13.86, *p* < 0.0005, Kendall’s W = 0.57) for during and after Ramadan in comparison with BR (*p* = 0.007 and *p* = 0.01 respectively). Thus, there was a significant effect of periods on scores for the sleep duration (test = 8.72, *p* = 0.01, Kendall’s W = 0.36) with higher scores during Ramadan in comparison with BR (*p* = 0.04). However, no-significant difference in the latency sleep (test = 3.5, *p* = 0.17, Kendall’s W = 0.14), sleep efficiency (test = 2, *p* = 0.36, Kendall’s W = 0.08), sleep disturbance (test = 4, *p* = 0.13, Kendall’s W = 0.16) or daytime dysfunction (test = 2.92, *p* = 0.23, Kendall’s W = 0.12) scores were observed.

## 4. Discussion

The purpose of the present study was to investigate the effect of a 25 min of nap opportunity on physical performance during the 5mSRT, feelings state, attention and the perception of fatigue before, during and after Ramadan observance in young physically active men. The main findings were that: (i) attention, TD and FI increased significantly after N25 in comparison with N0 during 10AR and 20AR and (ii) BD was significantly higher after N25 in comparison with N0 during BR and 10AR.

Under N0, TD and FI during the 5mSRT were unaffected by Ramadan observance, supporting the results of Boukhris et al. [11]. However, BD was significantly lower during ER compared to 10AR. This specific finding contradicts the results of Boukhris et al. [11], and it could be explained by the fact that in the present study sleep quality was negatively affected by Ramadan observance, which was not the case in the study of Boukhris et al. [11]. The decreased fractional contribution of carbohydrate to the diet was another possible contributor to the decline of BD during ER. BD (i.e., registered in all cases for the first 30-s repetition) is possibly related to alactic or adenosine triphosphate and phosphocreatine (ATP-PCr) capacity. The maintenance of TD during Ramadan observance could be related to the increased total fat intake (as previously suggested by Boukhris et al. [11]); increased fat-oxidation could retard the onset of fatigue through the sparing of muscle glycogen [42]. TD during the 5mSRT was determined mainly by the aerobic pathway, as the total duration of this physical exercise was more than 5 min. Additionally, in the present study, the lack of significant changes in RPE and feelings scores during Ramadan could explain the unchanged TD, supporting the conclusion of Boukhris et al. [11]. As reported by Boukhris et al. [19], the unchanged stress, sleep, fatigue, and muscle soreness scores during Ramadan observance further support the unchanged TD during the 5mSRT.

Many tactics have previously been suggested to reduce the negative effects of Ramadan observance (e.g., a tapering period [25], listening to motivational music [43]). We hypothesized that a 25-min nap opportunity might reduce perceptions of fatigue and the deterioration in physical and cognitive performance. Indeed, previous studies have reported that a 25-min nap opportunity benefits performance and perception of fatigue during the 5mSRT [30,31]. To the best of the authors’ knowledge, this is the first study investigating the effect of a nap opportunity on physical and cognitive performances during Ramadan. Although some positive effects were observed, a statistically significant impact was not reached for the effects of N25 on physical (i.e., performance during the 5-m shuttle run test) and cognitive performance (i.e., digit cancelation test) during Ramadan observance. Additionally, attention was unchanged in the present study after the N25 which could in part explain the unchanged physical performance. It is possible that if future studies use other physical tests that have been reported to be affected by Ramadan, e.g., repeated sprint [18], 30-s Wingate test [10,14,15], significant effect of the N25 will be observed. 

However, benefits were seen immediately after Ramadan. In the same way, previous studies showed that napping might improve physical [29,30,31,32,33,44] and cognitive [32,33] performance. The absence of a significant nap effect during Ramadan could be related to its duration or timing and/or to the extent of sleep perturbation incurred during Ramadan. Concerning the timing of the nap, Abdessalem et al. [31] noted that N25 at 14h00 and 15h00 improved the physical performance during the 5mSRT in comparison with N0; however, napping at 13h00 did not. Concerning the duration of the nap, Petit et al. [45] reported that a 20-min nap was insufficient to observe a significant effect during the 30-s Wingate test. Additionally, after a normal night’s sleep, Boukhris et al. [30] indicated that the improved physical performance during the 5mSRT was better after a 45 min as compared to a 25-min nap. Additionally, Hsouna et al. [32] noted that improvement in performance of the 5-jump test was seen only when the nap duration was over 35 min. Likewise, after a sleep deprivation night, Hammouda et al. [44] confirmed that the beneficial effects of a nap on repeated-sprint performances were observed after a 90 min but not after a 20-min nap opportunity. A sleep duration of less than 1 h probably provides insufficient time for the slow-wave stages of sleep to develop (i.e., period that has the greatest recuperative value) [3]. Future studies should examine both different durations and the timing of naps in relation to cognitive and physical performance disturbances during Ramadan observance.

On the other hand, the improvement of physical performance has been associated with a reduction of subjective fatigue [30,46], a reduction of sleepiness [29] and an improvement in alertness [46]. Therefore, the lack of a significant improvement of attention estimated by the digit cancellation test and a reduction of RPE after N25 during Ramadan observance could explain the unchanged physical performance in the present study.

The major limitation of the present work was the lack of objective measurement of the night and nap opportunity sleep (e.g., by the use of actigraphy or polysomnography). Thus, future research should record sleep during the experimental period using actigraphy or polysomnography. Additionally, the measurement of psychological differences between the periods of measurement could be important to support the findings of the present study.

## 5. Conclusions

Twenty-five minutes of nap opportunity was beneficial for physical and cognitive performance after Ramadan observance; however, naps of this duration were not sufficient to reach significant differences during the month of Ramadan.

## Figures and Tables

**Table 1:** Feeling scale, RPE and digit cancellation test scores (mean ± SE) recorded at 15 days before Ramadan (BR), during the first 10 days of Ramadan (FR), the last 10 days of Ramadan (ER), 10 days after Ramadan (10AR) and 20 days after Ramadan (20AR) after a no-nap (N0) or a 25-min of nap opportunity (N25) conditions.

PARAMETER	BR		FR		ER		10 AR		20 AR	
	N0	N25	N0	N25	N0	N25	N0	N25	N0	N25
Feeling scale (a.u)	0.33 ± 0.36	0.42 ± 0.5	−0.33 ± 0.31	0.75 ± 0.33+	−0.42 ± 0.29	0.08 ± 0.38	−0.17 ± 0.21	0.17 ± 0.21	−0.17 ± 0.21	0.33 ± 0.22+
RPE (a.u)	4.54 ± 0.21	4.83 ± 0.38	4.11 ± 0.26	3.83 ± 0.2¥	4.07 ± 0.2#	3.68 ± 0.18¥	3.92 ± 0.18#	3.57 ± 0.14¥	3.70 ± 0.16#¤	3.57 ± 0.16¥
Digit cancellation test (a.u)	65.8 ± 2.9	65.6 ± 3.3	62.2 ± 4.0	65.8 ± 3.8	62.4 ± 3.8	65 ± 3.7	61.8 ± 3.1	67.8 ± 3.3+	64.9 ± 3.2	71.3 ± 3.8+&

+: significant difference in comparison with N0; #: Significant difference in comparison with BR for N0; ¥: Significant difference in comparison with BR for N25; ¤: Significant difference in comparison with ER for N0; &: Significant difference in comparison with ER for N25.

**Table 2:** Dietary intake parameters (means ± SE) recorded 15 days before Ramadan (BR), during the first 10 days of Ramadan (FR), the last 10 days of Ramadan (ER), 10 days after Ramadan (10AR) and 20 days after Ramadan (20AR) under no-nap (N0) and 25-min nap opportunity (N25) conditions.

PARAMETER	BR	FR	ER	10 AR	20 AR
Energy intake (kJ/d)	11,436.3 ± 632.0	11,192.2 ± 457.4	10,198.5 ± 549.4	9989.3 ± 613.8	10,355.4 ± 517.3
Carbohydrates (%)	49.5 ± 1.6	44.3 ± 2.5	44.7 ± 2.1	52.3 ± 0.7@¶	52.3 ± 1.2@¶
Lipids (%)	35.8 ± 2.7	43.3 ± 2.1	40.9 ± 2.7	35.2 ± 1.0@	34.8 ± 1.4@
Proteins (%)	14.7 ± 2.4	12.5 ± 0.7	12.7 ± 0.6	12.5 ± 0.7	12.9 ± 0.7

@: Significant difference in comparison with ER; ¶: Significant difference in comparison with FR.

**Table 3:** PSQI questionnaire parameters (mean ± SE) recorded before Ramadan (BR), during Ramadan (DR) and after Ramadan (AR).

PSQI PARAMETER	BR	DR	AR
Subjective sleep quality (a.u)	0.92 ± 0.17	2 ± 0.17♦	1.75 ± 0.19♦
Sleep latency (a.u)	0.67 ± 0.2	1 ± 0.16	0.92 ± 0.13
Sleep duration (a.u)	0.5 ± 0.21	1.17 ± 0.24♦	1.08 ± 0.26
Sleep efficiency (a.u)	0.08 ± 0.07	0.17 ± 0.1	0.08 ± 0.07
Sleep disturbance (a.u)	0.5 ± 0.13	0.63 ± 0.13	0.67 ± 0.13
Daytime dysfunction (a.u)	0.33 ± 0.17	0.58 ± 0.2	0.33 ± 0.13
Global score PSQI (a.u)	3 ± 0.52●	6 ± 0.5	5 ± 0.44●

**♦**: Significant difference in comparison with BR; ●: Significant difference in comparison with DR.
